# *Pseudomonas aeruginosa* Alters Critical Lung Epithelial Cell Functions through Activation of ADAM17

**DOI:** 10.3390/cells11152303

**Published:** 2022-07-26

**Authors:** Ahmad Aljohmani, Noah Niklas Andres, Daniela Yildiz

**Affiliations:** Institute of Experimental and Clinical Pharmacology and Toxicology, PZMS, ZHMB, Saarland University, 66421 Homburg, Germany; ahmad.aljohmani@uks.eu (A.A.); noah1andres@gmail.com (N.N.A.)

**Keywords:** proteolysis, metalloproteinase, lung infection, junctional molecules, regeneration, exosomes

## Abstract

Severe epithelial dysfunction is one major hallmark throughout the pathophysiological progress of bacterial pneumonia. Junctional and cellular adhesion molecules (e.g., JAMA-A, ICAM-1), cytokines (e.g., TNFα), and growth factors (e.g., TGFα), controlling proper lung barrier function and leukocyte recruitment, are proteolytically cleaved and released into the extracellular space through a disintegrin and metalloproteinase (ADAM) 17. In cell-based assays, we could show that the protein expression, maturation, and activation of ADAM17 is upregulated upon infection of lung epithelial cells with *Pseudomonas aeruginosa* and Exotoxin A (ExoA), without any impact of infection by *Streptococcus pneumoniae*. The characterization of released extracellular vesicles/exosomes and the comparison to heat-inactivated bacteria revealed that this increase occurred in a cell-associated and toxin-dependent manner. Pharmacological targeting and gene silencing of ADAM17 showed that its activation during infection with *Pseudomonas aeruginosa* was critical for the cleavage of junctional adhesion molecule A (JAM-A) and epithelial cell survival, both modulating barrier integrity, epithelial regeneration, leukocyte adhesion and transepithelial migration. Thus, site-specific targeting of ADAM17 or blockage of the activating toxins may constitute a novel anti-infective therapeutic option in *Pseudomonas aeruginosa* lung infection preventing severe epithelial and organ dysfunctions and stimulating future translational studies.

## 1. Introduction

Pneumonia is a serious, life-threatening lung infection mainly characterized by an inflammation of the alveoli leading to various pathophysiologies such as respiratory failure, acute respiratory distress syndrome (ARDS) and sepsis [1]. Bacterial pneumonia is broadly divided into hospital-acquired pneumonia and community-acquired pneumonia which are mostly caused by the gram-negative bacteria *Pseudomonas aeruginosa* (*P. aeruginosa*) [2] and the gram-positive bacteria *Streptococcus pneumoniae* (*S. pneumoniae*) [3], respectively. The inhaled pathogens exhibit several challenges mediated by a collaborative effort of several cell types including the lung epithelium, dendritic cells, T cells, macrophages, and neutrophils [4]. As the first line of defense, the lung epithelium acts as a tight barrier against invading pathogens and as an alarm for the innate and adaptive immune systems [5]. In worst cases, the aspirated pathogens are able to cross the lung barrier further disseminating through these defenses and predisposing to a number of inflammatory and clinical manifestations.

Epithelial cells play a significant role throughout the steps of infection by secretion of complement proteins that bind infectious agents and promote phagocytosis [6]. In addition, epithelial cells promote leukocyte recruitment through the release of cytokines and chemokines, predominantly IL-1β and IL-8 upon activation of their toll-like receptors (TLR) [6]. *P. aeruginosa* triggers this inflammatory reaction through interaction with TLR2 and TLR4 [7,8] or through cellular adhesion via its flagella and type 4 pili [4]. Furthermore, *P. aeruginosa* releases several virulence factors, mainly derived from type 2 and type 3 secretion systems (T2SS and T3SS) [9]. T3SS-derived toxins including the four exoenzymes ExoS, ExoT, ExoY and ExoU have been shown to regulate lung permeability through stress fiber formation, cytoskeletal rearrangement, and disruption of cell-cell junctions [10]. The most prominent member of T2SS-derived toxins is ExoA. ExoA-producing strains show a much higher virulence compared to ExoA-deficient mutants, which can be prevented by vaccination with inactivated ExoA [11]. This detrimental action is at least in parts based on lung epithelial barrier disruption, which we could link to activation of a disintegrin and metalloproteinase (ADAM) 10 in a recent study [12,13]. 

ADAM proteases are type 1 transmembrane proteins regulating, for example, cell proliferation, cell migration, cell adhesion and proteolysis [14]. Proteolytically active family members, including ADAM17, are characterized by the regulated release of soluble ectodomains close to the plasma membrane (shedding) as central regulators of several inflammatory and infectious diseases (for review, see [15]). Substrates for ADAM proteases include, but are not limited to, growth factors (e.g., transforming growth factor (TGFα) and epidermal growth factor (EGF)), cytokines and chemokines (e.g., fractalkine and TNF) as well as their receptors (e.g., TNFR, IL-6R), and adhesion molecules (E- and VE-Cadherin) [14]. It has been shown that ADAM17 is capable to cleave several cell adhesion molecules (CAM) (e.g., VCAM-1 and ICAM-1) and junctional adhesion molecule A (JAM-A), influencing leukocyte recruitment and endothelial damage [16,17]. In addition, ADAM17 inhibition increased bacterial phagocytosis by leukocytes in a cell-autonomous manner [18]. Therefore, we hypothesized that the functional impact of ADAM17 may not be limited to leukocytes and endothelial cells but could be also essential for epithelial cell functions during infection.

In the present study, we could show a pathogen-dependent regulation of ADAM17 in alveolar epithelial cells during infection with *P. aeruginosa* and *S. pneumoniae*. Infection with *P. aeruginosa* or stimulation with ExoA as representative of its major virulence toxins increased ADAM17 protein expression and activity, whereas no changes upon infection with *S. pneumoniae* were observed. This cell-associated action of ADAM17 led to enhanced protein permeability, decreased wound closure, and reduced transepithelial leukocyte migration linked to cleavage of JAM-A. Furthermore, inhibition or lack of ADAM17 in epithelial cells increased epithelial cell survival and leukocyte adhesion, promoting trans-epithelial migration and barrier integrity. Thus, inhibition of ADAM17 activity may be a promising novel anti-infective treatment option in lung infection to prevent barrier disruption and increase the clearance of the invading pathogens. Therefore, site-specific treatment strategies by the blocking of either ADAM17 activity or the activating toxins should be further investigated in translational in vivo studies.

## 2. Materials and Methods

### 2.1. Antibodies, Chemokines and Inhibitors

Rabbit anti-human ADAM17 (C-terminus), and mouse anti-human CD9 were obtained from Invitrogen (Frankfurt, Germany). Rabbit anti-human GAPDH was purchased from Santa Cruz Biotech (Dallas, TX, USA), mouse anti-human JAM-A and mouse anti-human Flotilin-1 from BD Biosciences (Heidelberg, Germany), and peroxidase-conjugated anti-mouse and anti-rabbit IgG secondary antibodies from GE Healthcare (Chicago, IL, USA). TAPI-1, active-site inhibitor of ADAM17, was from Merck Millipore (Darmstadt, Germany) and human CCL2, a monocyte chemoattractant, was from Peprotech (Rocky Hill, NJ, USA). For more details see Table 1.

### 2.2. Bacteria Preparation

*Pseudomonas aeruginosa* (*P. aeruginosa*, PA103 strain) and *Streptococcus pneumoniae* (*S. pneumoniae*, R6 strain) were grown as described earlier [12]. Briefly, the bacteria were incubated in Todd–Hewitt–Bouillon (THB) medium at 37 °C at 150 rpm until reaching the exponential phase. Consequently, bacterial cultures were centrifuged and prepared in PBS for stimulation with a multiplicity of infection (MOI) of 5. *P. aeruginosa* heat in-activation was done by incubating the bacteria in the heatblock for 40 min at 70 °C [19]. 

### 2.3. Cell Culture

RPMI1640 (+10% FCS), DMEM (+10% FCS) and MV2 (PromoCell, Heidelberg, Germany) medium were used to culture THP-1, A549 and Human small airway epithelial cells (HSAEpC), respectively [17]. In each experiment (as indicated in the figure legends) the cells were pre-incubated with TAPI-1 (10 µM) or DMSO (0.1%) for 30 min. Subsequently, the cells were infected with either *P. aeruginosa* or *S. pneumoniae* or stimulated with Exotoxin A (ExoA, Merck Millipore, Darmstadt, Germany) as indicated in the figure legends [12,17,20].

### 2.4. Western Blot (WB)

Western blot analysis was carried out as described [12]. Cell lysates were obtained by incubating the cells with lysis buffer (20 mM Tris·HCl, 150 mM NaCl, 1% Triton X-100, 1 mM EDTA, 1 mM Na3VO4, 1 mM PMSF, 10 mM 1,10- phenanthroline monohydrate, 1x Complete inhibitor), and protein concentration was determined using the bicinchoninic acid assay (BCA) kit from Thermo Fisher (Karlsruhe, Germany). Tris-glycine gels were used to separate the proteins (equal amounts loaded per lane) according to the molecular weight before being transferred to a nitrocellulose membrane (GE Healthcare Life Sciences, Freiburg, Germany). Membranes were incubated overnight with the desired primary antibody at 4 °C, and the chemiluminescence substrate (PerkinElmer, Waltham, MA, USA) was added to analyze the chemiluminescence signals using the image analyzer LAS3000 (Fujifilm, Tokyo, Japan). Densitometric analysis was performed by AIDA Image Analysis software 4.27.039 (Elysia-raytest, Straubenhardt, Germany).

### 2.5. Lentiviral Transduction

Lentivirus production and cell transduction were performed as described earlier [12]. The production of lentivirus was performed by transfecting HEK293T cells as described [20]. The targeting sequences for ADAM17 were AGGAAAGCCCTGTACAGTA (ADAM17-KD1) and GAAACAGAGTGCTAATTTA (ADAM17-KD2). Furthermore, CCGTCACATCAATTGCCGT sequence was used as scramble (scr) control. 2 × 10^5^ A549 cells were transduced with lentivirus for 24 h in the presence of polybrene (4 µg/mL). The efficiency of the transduction was controlled by the GFP signal and the knockdown by WB.

### 2.6. Catalytic Activity Assay

Lipofectamine™ 3000 (Thermo Fisher, Karlsruhe, Germany) was used to transfect A549 cells with the plasmid of TGF-alpha (TGF-a) coupled with alkaline phosphatase (AP) at the N-terminus. After stimulation (see figure legends), the activity of alkaline phosphatase was analyzed in both cell lysate and the medium using a Genios fluorescence reader (Tecan, Grödig, Austria) as described [21]. Stimulation with PMA in the presence or absence of 10 µM TAPI-1 for 1 h served as assay control [21]. 

### 2.7. Exosome Preparation

2 × 10^7^ A549 cells were cultured in a serum-free medium followed by *P. aeruginosa* infection and exosome preparation as described previously [12]. Briefly, the medium was assigned for deferential centrifugation at 300× *g*, 1000× *g*, 10,000× *g* and finally 100,000× *g* (for extracellular vesicle (EV) collection) at 4 °C using an ultracentrifuge with rotor Type Ti50.2 from Beckman Coulter GmbH (Krefeld, Germany). The resulting pellet of each centrifugation step was lysed in SDS buffer or resuspended in ice-cold PBS for exosome purification. Subsequently, the resulted EVs were further separated according to their density by centrifugation at 100,000× *g* for 16 h at 4 °C using a sucrose gradient of different concentrations (2, 1.3, 1.16, 0.8, 0.5 and 0.25 M) followed by further centrifugation for each resulted layer at 150,000× *g* for 4 h at 4 °C. The pellet from each fraction was lysed in an SDS buffer and analyzed by WB [12]. 

### 2.8. Transepithelial Permeability Assay

The transepithelial protein permeability assay was performed as described earlier [12]. A549 cells were grown until confluence in 5 µm pores transwell filters (Corning, Amsterdam, The Netherlands) coated with collagen G (40 µg/mL Biochrom, Germany). After the indicated treatment, the upper chamber’s medium was removed and replaced by a suspension of 70-kDa TRITC-dextran (1 mg/mL) and FITC-albumin (0.25 mg/mL, Sigma-Aldrich) in PBS supplemented with 0.2% BSA. After 90 min, the fluorescence intensity of TRITC-dextran and FITC-albumin in the lower chamber was quantified using the Genios fluorescence reader [12].

### 2.9. Transepithelial Migration

The transepithelial migration assay was performed using transwell chambers (Corning, Amsterdam, The Netherlands) as described in detail in [12]. Briefly, A549 cell monolayers were treated as indicated. Subsequently, 2 × 10^5^ THP-1 cells were added to the upper chamber to assess random migration (absence of CCL2 in the lower chamber) and chemotaxis (presence of 3 nM CCL2 in the lower chamber). The number of transmigrated THP-1 cells was analyzed by determination of endogenous β-glucoronidase activity in the lower well as described before [22].

### 2.10. Wound Closure Assay (Scratch Assay)

96-well plates were coated with collagen G and A549 cells were seeded and grown until confluence. Subsequently, cellular proliferation was blocked by incubation with mitomycin (5 µg/mL) for 2 h followed by the treatment indicated in the figure legend. An automated scratch was done in each well (BioTec autoscratch, Highland Park, IL, USA), and wound/scratch closure was tracked and quantified using the Lionheart (FX) Automated Microscope system (BioTec, Highland Park, IL, USA) with Gen5 image Prime software 3.05.11 (BioTek, Highland Park, Winooski, VT, USA). 

### 2.11. Adhesion Assay

1 × 10^5^ A549 cells were seeded on 24-well plates and grown to confluence. THP-1 cells were fluorescently labeled with calcein-AM (1 mM) for 30 min. After infection of the A549 monolayer, the cells were washed with warm PBS followed by the addition of 5 × 10^5^ fluorescently labeled THP-1 cells. Subsequently, the plate was centrifuged for 3 min at 300 g, washed three times with warm PBS, and the fluorescence of the adhered THP-1 cells was measured at 480-nm excitation and 520-nm emission wavelength using the Lionheart (FX) Automated Microscope system. 

### 2.12. Survival Assay

2 × 10^5^ A549 cells were seeded on 12-well plates and grown to confluence. A549 cells were stained using the cell-permeant nuclear stain NucRed™ Live 647 (Thermo Fisher, Karlsruhe, Germany) for 15 min, washed three times with PBS and infected with *P. aeruginosa* for 4 h in the presence of the plasma membrane impermeable nucleic acid stain SYTOX™ green (200 nM, Thermo Fisher, Karlsruhe, Germany). The fluorescence intensity was measured every 30 min (480/520-nm excitation/emission wavelength for SYTOX™ green and 638/686-nm excitation/emission wavelength for NucRed™ Live 647) using the Lionheart (FX) Automated Microscope system. The fluorescence signals were quantified by applying an automated primary mask recognizing the green fluorescent cells. 

### 2.13. Statistical Analysis

Quantitative data are shown as mean + SD from at least three independent experiments, and analysis was performed using GraphPad PRISM 9.0 (GraphPad Software, La Jollla, CA, USA) with a *p*-value < 0.05 regarded as significant. The used statistical analyses are detailed in the figure legends.

## 3. Results

### 3.1. Regulation of ADAM17 upon Infection of Lung Epithelial Cells Occurs in a Pathogen-Dependent Manner

**Figure 1:**
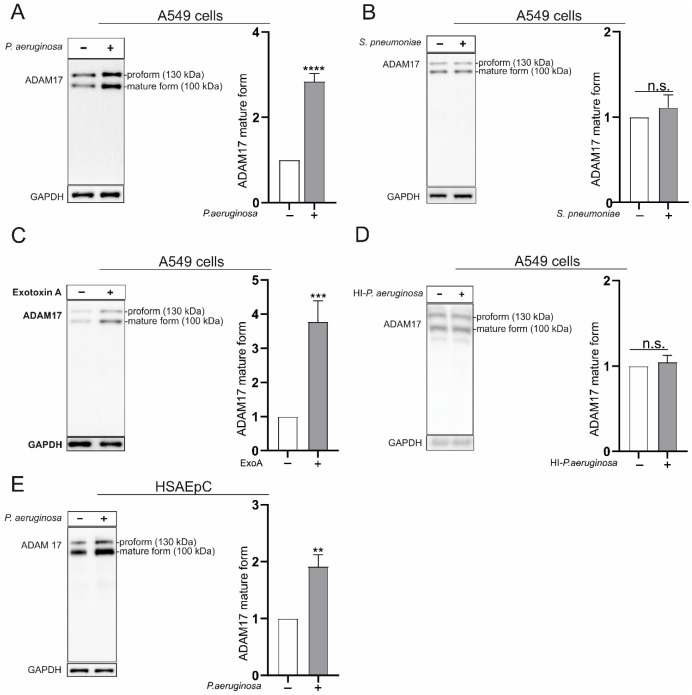
Regulation of a disintegrin and metalloproteinase (ADAM) 17 protein expression and maturation in bacterial infection. A549 (**A**–**D**) or human small airway epithelial cells (HSAEpC) (**E**) were grown until confluence. Cells were either left unstimulated or infected with either *Pseudomonas aeruginosa* (*P. aeruginosa,* multiplicity of infection (MOI) 5) (**A**,**E**) or *Streptococcus pneumoniae* (*S. pneumoniae*, MOI 5) (**B**), stimulated with Exotoxin A (ExoA, 100 ng/mL) (**C**) or infected with heat-inactivated (HI) *P. aeruginosa* (MOI 5) (**D**). Samples were taken after 4 h of incubation, and the protein expression and maturation of ADAM17 were analyzed through Western blot with an antibody against the C-terminal domain of ADAM17 in comparison to the protein expression of glyceraldehyde-3-phosphat dehydrogenase (GAPDH, internal loading control). Densitometry was used to evaluate band intensities, which were further normalized to the expression of the unstimulated cells. Quantitative data are shown as means + SD of three independent experiments. Asterisks indicate significance difference to the control calculated using two-tailed two samples *t*-test (** *p* < 0.01, *** *p* < 0.001, **** *p* < 0.0001, n.s. not significant).

Changes in barrier integrity of the alveolar epithelium are key factors in the manifestations of lung pneumonia and the development of systemic side effects. Based on the substrate spectrum, a contribution of disintegrin and metalloproteinase (ADAM) 17 to these processes seems quite feasible. To obtain the first hint, the regulation of cell-associated ADAM17 was investigated in A549 cells (alveolar adenocarcinoma cells) and human small airway epithelial cells (HSAEpC) upon infection with different bacteria. In A549 cells, the Gram-negative bacterium *Pseudomonas aeruginosa* (*P. aeruginosa*) induced protein expression and maturation of ADAM17, shown by a stronger expression (densitometric analysis) of the 100 kDa mature form and the 130 kDa pro-form of ADAM17 after 4 h of infection (Figure 1A). 

On the other hand, neither protein expression nor maturation of ADAM17 was changed upon infection with the Gram-positive bacterium *Streptococcus pneumoniae* (*S. pneumoniae*, Figure 1B). Besides the effect of the pathogen itself, solely the released toxins and virulence factors could regulate protease expression. Therefore, we used the stimulation with exotoxin A (ExoA) as one representative of the secreted virulence factors/toxins released by *P. aeruginosa* [23]. Interestingly, stimulation with ExoA initiated the same changes in protein expression and maturation of ADAM17 as observed upon *P. aeruginosa* infection (Figure 1C). To evaluate the effect of the bacterial particle itself, we subjected *P. aeruginosa* to mild heat inactivation (40 min, 70 °C) keeping its structural properties but disturbing the structure and activity of the secreted toxins [12]. In contrast to living bacteria and ExoA, heat-inactivated *P. aeruginosa* had no impact on the protein expression or maturation of ADAM17 (Figure 1D). The same regulation pattern of ADAM17 was observed in HSAEpC as primary human lung epithelial cell (*P. aeruginosa* Figure 1E, ExoA Figure S1A and *S. pneumoniae* Figure S1B). Thus, these findings suggest a differential regulation of epithelial ADAM17 in a pathogen- and toxin-dependent mode during bacterial infection.

### 3.2. ADAM17 Activation and Shedding Activity Are Stimulated by P. aeruginosa and ExoA in a Cell-Associated Manner

**Figure 2:**
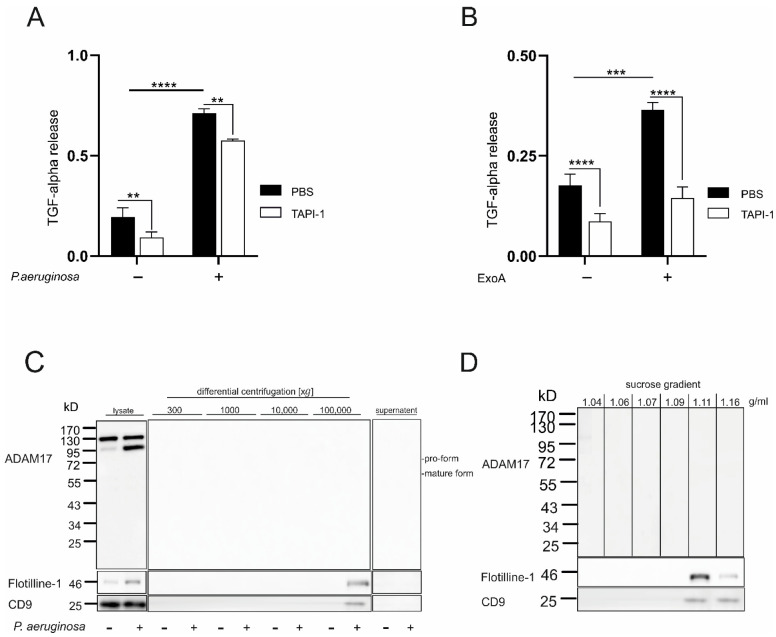
Activation and exosomal release of ADAM17 by *P. aeruginosa* and ExoA. (**A**,**B**) A549 cells expressing plasmid-encoded TGF-alpha (TGF-a) coupled with alkaline phosphatase (AP, N-terminal) were pre-incubated for 30 min with the ADAM17 inhibitor TAPI-1 (10 µM) or DMSO (0.1%). Subsequently, cells were left untreated, challenged with *P. aeruginosa* (**A**, MOI 5) or ExoA (**B**, 100 ng/mL) for 4 h. The activity of alkaline phosphatase was analyzed in both cell lysate and the medium as an indicator for TGF-α cleavage and release. Quantitative data are shown as means + SD of three independent experiments. Asterisks indicate significance among treated cells calculated using two-way ANOVA and Tukey post-test (** *p* < 0.01, *** *p* < 0.001, **** *p* < 0.0001). (**C**,**D**) A549 cells were either left untreated or challenged with *P. aeruginosa* (MOI 5) followed by a differential centrifugation of the supernatant (300, 1000, 10,000, 100,000 g). The resulting pellet from each centrifugation step was lysed with SDS buffer and analyzed by Western blot (**C**) developing against the C-terminus of ADAM17, Flotiline-1 and CD9 (exosome markers). The pellet obtained from the 100,000 g centrifugation in step C, containing extracellular vesicles (EVs), was used to further separate the EVs according to their density. The resulted fractions were analyzed by Western blot and probed against ADAM17, Flotiline-1 and CD9 (**D**). Representative blots of at least three independent experiments are shown. No ADAM17 expression in EVs/exosomes could be observed.

To clarify whether these changes in the regulation of ADAM17 are also reflected on the functional level, the activity of ADAM17 in response to *P. aeruginosa* and ExoA was investigated using a substrate cleavage assay, in which alkaline phosphatase (AP)-tagged TGF-alpha (AP-TGF-α) was used as a described substrate for ADAM17 [24]. Based on the same regulation pattern in A549 cells and primary lung epithelial cells, the cell line was used due to the higher capacity for genetic modification and transient protein expression. AP activity measured in the cell supernatant, reflecting the release of soluble TGF-α, was significantly increased 4 h after infection with *P. aeruginosa* in comparison to control. Pre-treatment with the ADAM17 inhibitor TAPI-1 significantly decreased the AP-TGF-α release (Figure 2A and Figure S1C). In addition, TAPI-1 decreased the basal AP-TGF-α release observed in control cells. Further, ExoA induced a significant release of AP-TGF-α to the supernatant, while this release was notably reduced upon pre-incubation with TAPI-1 (Figure 2B and Figure S1D). In general, the increase in ADAM17 activity upon ExoA stimulation was weaker than observed upon infection with *P. aeruginosa*, but comparable to PMA control stimulation (Figure S1E). Although no changes were seen on the expression/maturation level after infection with *S. pneumoniae*, we tested if changes might only occur on the activation level. However, the activity of ADAM17 was not changed upon infection with *S. pneumoniae* in comparison to non-stimulated cells (Figure S1F). It was recently reported that *P. aeruginosa* triggered the release of ADAM10 on exosomes [12]. To investigate if this may also hold true for ADAM17, we investigated extracellular vesicles and further fractionized exosomes from infected and non-infected A549 cells for the presence of ADAM17. Interestingly, ADAM17 could not be detected in extracellular vesicles or exosomes (Figure 2C,D). Thus, infection with *P. aeruginosa* leads to an increase in cell-associated ADAM17 expression, maturation and shedding activity.

### 3.3. Epithelial ADAM17 Mediates P. aeruginosa Induced Protein Permeability

**Figure 3:**
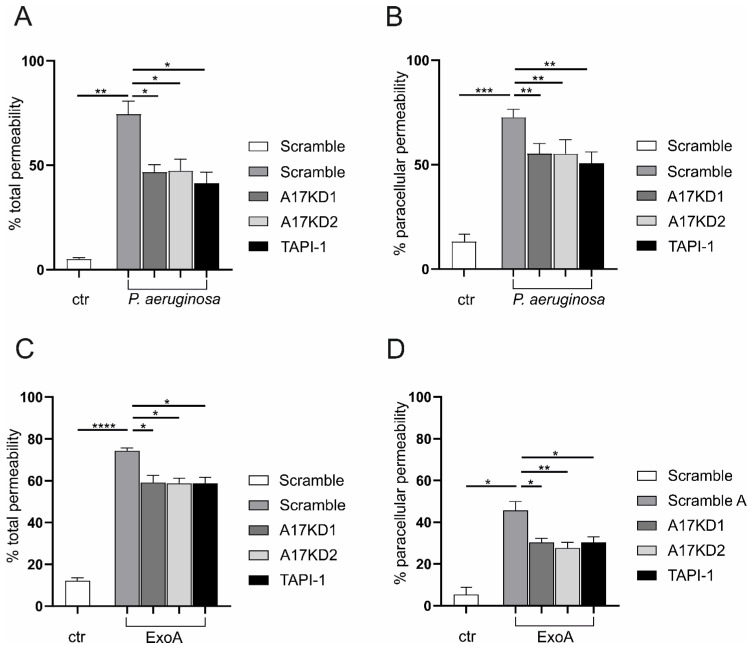
ADAM17 induces protein permeability during *P.* *aeruginosa* infection. shRNA sequences against ADAM17 (A17KD1 or A17KD2), delivered by lentiviral particles, were used to mediate ADAM17 deprivation in A549 cells while an unspecific shRNA sequence served as scramble control (scr). (**A**–**D**) Cells were seeded on transwells until formation of a monolayer and pre-incubated with DMSO (0.1%) or TAPI-1 (10 µM). Subsequently, cells were either left untreated, challenged with *P. aeruginosa* (**A**/**B**, MOI 5) or stimulated with ExoA (**C**/**D**, 100 ng/mL) for 4 h in the absence of presence of 10 µM TAPI-1. Subsequently, the upper chamber’s medium was replaced by 70-kDa TRITC-dextran and FITC-albumin suspension (1 mg/mL and 0.25 mg/mL, respectively, in PBS supplemented with 0.2% BSA), and the permeability was evaluated by the diffusion of the suspension to the lower chamber. The percentages of both total and paracellular permeabilities are shown relative to an empty transwell (maximal permeability, 100%). Quantitative data are shown as mean + SD of three independent experiments. Asterisks indicate significance among treated cells calculated using one-way ANOVA and Tukey post-test (* *p* < 0.05, ** *p* < 0.01, *** *p* < 0.001, **** *p* < 0.0001). In both cases, we observed a significant reduction in protein permeability either upon infection with *P. aeruginosa* or stimulation with ExoA. Thus, the lack of ADAM17 induction may improve barrier function.

One of the main functions of the tight barrier is to prevent the free diffusion of ions and small solutes along the paracellular pathway [25]. Barrier integrity is physiologically maintained by a collaborative interaction of a wide range of junction and adhesion molecules [26], including several substrates of ADAM17 [27]. Therefore, monolayers of A549 cells in transwells were either infected with *P. aeruginosa* or stimulated with ExoA and evaluated for protein permeability using TRITC-dextran (paracellular permeability) and FITC-albumin (total permeability including paracellular and transcellular permeability) as tracers, respectively. Both paracellular and total protein permeabilities were significantly increased upon infection with *P. aeruginosa* or stimulation with ExoA (Figure 3A–D). Interestingly, *P. aeruginosa* led to a higher increase in paracellular permeability than ExoA as a single virulence factor (Figure 3B,D). To specifically address ADAM17, we used either pharmacological inhibition by TAPI-1 or gene silencing/knockdown by lentiviral delivery of ADAM17-specific shRNAs (control of knockdown efficiency by Western blot, see Figure S2A). 

### 3.4. ADAM17 Inhibition Improves Epithelial Wound Healing by Reduced Shedding of Junctional Molecules

**Figure 4:**
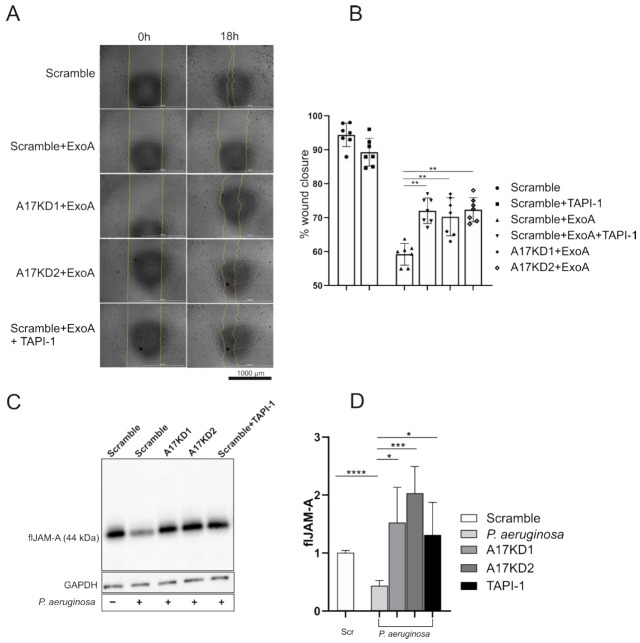
Impact of ADAM17 on epithelial regeneration. shRNA sequences against ADAM17 (A17KD1 or A17KD2), delivered by lentiviral particles, were used to mediate ADAM17 deprivation in A549 cells while an unspecific shRNA sequence served as scramble control (scr). Cells were grown to confluence and preincubated with either DMSO (0.1%) or TAPI-1 (10 µM) for 30 min. (**A**,**B**) Cells were treated with mitomycin (5 µg/mL) for 2 h to avoid cell proliferation and then either challenged for 4 h with ExoA (100 ng/mL) or left untreated. Subsequently, the stimulant was removed, an automated scratch was performed and the wound closure was monitored for 24 h using a live cell imaging system. The percentage of wound closure was calculated relative to a fully closed wound. Example images are shown in (**A**). (**C**,**D**) Cells were challenged with *P. aeruginosa* for 4 h or left untreated. After 4 h, the cells were lysed and the expression of JAM-A was analyzed by Western blot using an antibody against the N-terminal domain (extracellular part). GAPDH served as a loading control. An exemplary Western blot is shown in (**C**). Quantitative data are shown as mean + SD (n = 7 in (**B**), n = 5 in (**D**)). Asterisks indicate significance among treated cells calculated using one-way ANOVA and Tukey post-test (**A**,**B**) or two-tailed two samples *t*-test for (**C**,**D**) (* *p* < 0.05, ** *p* < 0.01, *** *p* < 0.001, **** *p* < 0.0001).

Damage to the lung epithelium is a hallmark of severe pneumonia. To investigate the functional consequences of ExoA-mediated activation of ADAM17 for epithelial damage and epithelial regeneration, a scratch wound closure assay was performed. Stimulation with ExoA significantly impaired the wound closure after 18 h compared to non-stimulated control cells by appr. 45% (Figure 4A,B). Notably, pharmacological inhibition of ADAM17 by TAPI-1 or gene silencing/knockdown improved wound closure by appr. 25%. 

Junctional molecules are responsible for cell-cell interactions as well as cell-extracellular matrix interactions, maintaining the stability of the barrier and the layer integrity [25]. Thus, the shedding of these molecules upon infection could impair epithelial regeneration. Therefore, we analyzed the effect of infection with *P. aeruginosa* and stimulation with ExoA, respectively, on the cellular presence of junctional adhesion molecule-A (JAM-A). Both stimulation with ExoA (Figure S2B,C) and infection with *P. aeruginosa* (Figure 4C,D) induced shedding of JAM-A indicated by a decrease in the full-length and cell-associated protein (44 kDa). This process was completely inhibited upon pharmacological inhibition or gene silencing/knockdown of ADAM17. Thus, prevention of junction molecule cleavage by ADAM17 inhibition may prevent disruption of lung epithelial integrity.

### 3.5. ADAM17 Enhances Transepithelial Migration through Enhanced Adhesion and Improvement of Cell Survival

The inflammatory process starts with a series of events including leukocyte recruitment, rolling and adhesion followed by transmigration. Most of these steps are highly regulated by several shedding events mediated by ADAM17 [14]. Therefore, we examined the impact of ADAM17 activity on transepithelial migration of monocytic cells (THP-1 cells) during infection with *P. aeruginosa*. Infection with *P. aeruginosa* induced not only a significant increase in the transepithelial migration of THP-1 cells in the presence (seven-fold) but also in the absence (five-fold) of the monocyte chemoattractant CCL2. 

**Figure 5:**
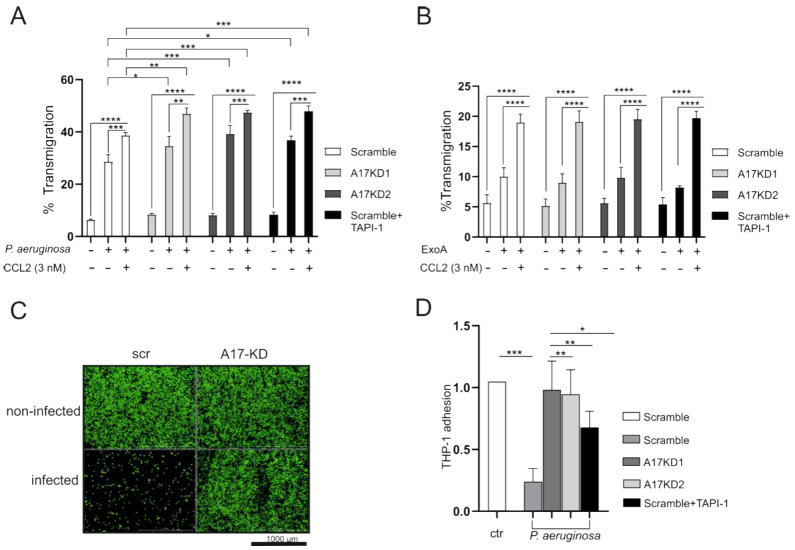
ADAM17 regulates THP-1 cells transepithelial migration and adhesion during *P. aeruginosa* infection. shRNA sequences against ADAM17 (A17KD1 or A17KD2), delivered by lentiviral particles, were used to mediate ADAM17 deprivation in A549 cells while an unspecific shRNA sequence served as scramble control (scr). (**A**,**B**) Cells were seeded on transwells until formation of a monolayer and pre-incubated with DMSO (0.1%) or TAPI-1 (10 µM). Subsequently, cells were left untreated, challenged with *P. aeruginosa* (A, MOI 5) or stimulated with ExoA ((**B**), 100 ng/mL) for 4 h in the absence or presence of 10 µM TAPI-1. The random and the chemotactic (against 3 nM CCL2) transepithelial migration were evaluated by the addition of 2 × 10^5^ THP-1 cells to the upper chamber. The number of transmigrated THP-1 cells was analyzed after 45 min by evaluation of endogenous β-glucoronidase activity in the lower well. (**C**,**D**) Cells were challenged with *P. aeruginosa* for 4 h or left untreated. Subsequently, 5 × 10^5^ fluorescently labeled THP-1 cells were added, centrifuged at 300× *g* for 3 min and washed with warm PBS before measuring the fluorescence intensity of the adhered cells. Quantitative data are shown as mean + SD of three independent experiments. Asterisks indicate significance as indicated by lines calculated using one-way ANOVA and Tukey post-test (* *p* < 0.05, ** *p* < 0.01, *** *p* < 0.001, **** *p* < 0.0001).

Interestingly, inhibition of ADAM17 by TAPI-1 or gene silencing/knockdown significantly increased the random and the CCL2-induced transepithelial migration of THP-1 cells (Figure 5A). However, ExoA stimulation led to a weaker induction of transmigration (2- to 4-fold) with no effect of ADAM17 inhibition or gene silencing (Figure 5B). One step up in the cascade, we investigated the role of ADAM17 on THP-1 cell adhesion during infection with *P. aeruginosa*. Infection with *P. aeruginosa* highly reduced THP-1 cell adhesion to A549 cells in comparison to non-infected cells. Notably, inhibition of ADAM17 by TAPI-1 or gene silencing/knockdown almost normalized THP-1-A549 cell adhesion (Figure 5C,D). 

**Figure 6:**
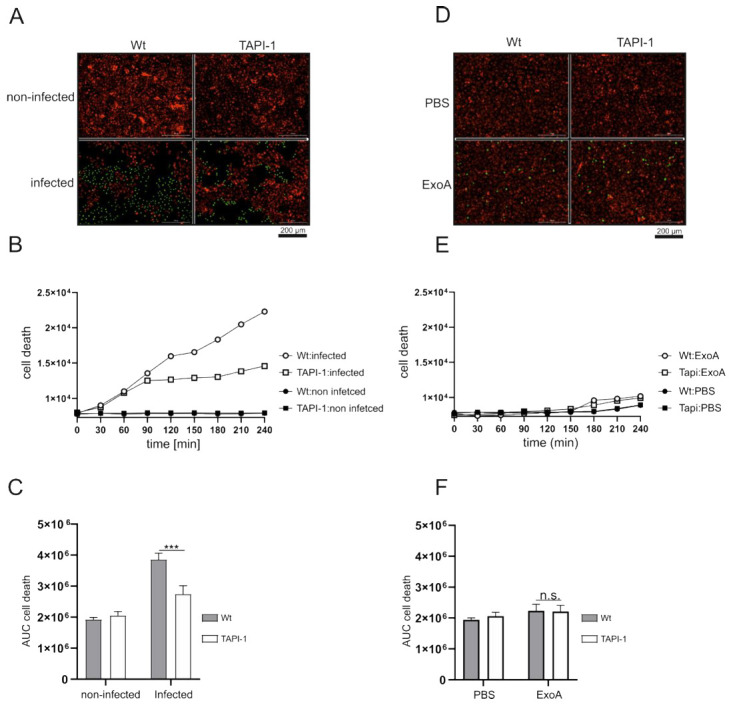
Reduced ADAM17 activity increases epithelial cell survival. (**A**–**F**) A549 cells were grown until confluence, stained with the cell membrane permeable dye NucRed™ Live 647 for 15 min and either left untreated or challenged with *P. aeruginosa* (A-C, MOI 5) or stimulated with ExoA (**D**–**F**, 100 ng/mL) in the presence of the cell membrane impermeable SYTOX™ green. SYTOX™ green fluorescence was evaluated every 30 min from each cell. Quantitative data are shown as means + SD of three independent experiments (**A**/**D**, representative image; **B**/**E**, fluorescence intensity over time of one experiment; **C**/**F**, area under the curve (AUC) of SYTOX™ green fluorescence over time of three independent experiments). Asterisks indicate significance among treated cells calculated using two-way ANOVA and Tukey post-test (*** *p* < 0.001, n.s. not significant).

Reduced adhesion and transepithelial migration may be also caused by the differential survival of the lung epithelial cells. Therefore, A549 cells were stained with SYTOX™ green (nucleic acid stain, cell membrane impermeable) and NucRed™ (nuclear stain, plasma membrane permeable) followed by infection with *P. aeruginosa* or stimulation with ExoA and evaluation of cell death by live-cell imaging. Infection with *P. aeruginosa* induced a strong increase in cell death, indicated by an increase in green and a decrease in red cells. Interestingly, inhibition of ADAM17 by TAPI-1 improved the A549 cell survival over time (Figure 6A–C). ExoA stimulation, however, had only a marginal effect on cell survival with no additional effect by pharmacological inhibition of ADAM17 (Figure 6D–F). Thus, besides junctional and adhesion molecule shedding, increased cell survival upon ADAM17 inhibition may explain enhanced adhesion and subsequently enhanced transepithelial migration of THP-1 cells during infection by *P. aeruginosa*. 

## 4. Discussion

Our present study shows that ADAM17 critically contributes to lung epithelial cell functions such as protein permeability and transepithelial migration through changes in junctional adhesion molecule shedding, leukocyte adhesion and cell survival. Importantly, the activation and function of ADAM17 occurred in a pathogen-dependent and cell-associated manner. Thereby, we provide further evidence for ADAM17 as a suitable target for novel anti-infective treatment strategies for airway diseases.

**Figure 7:**
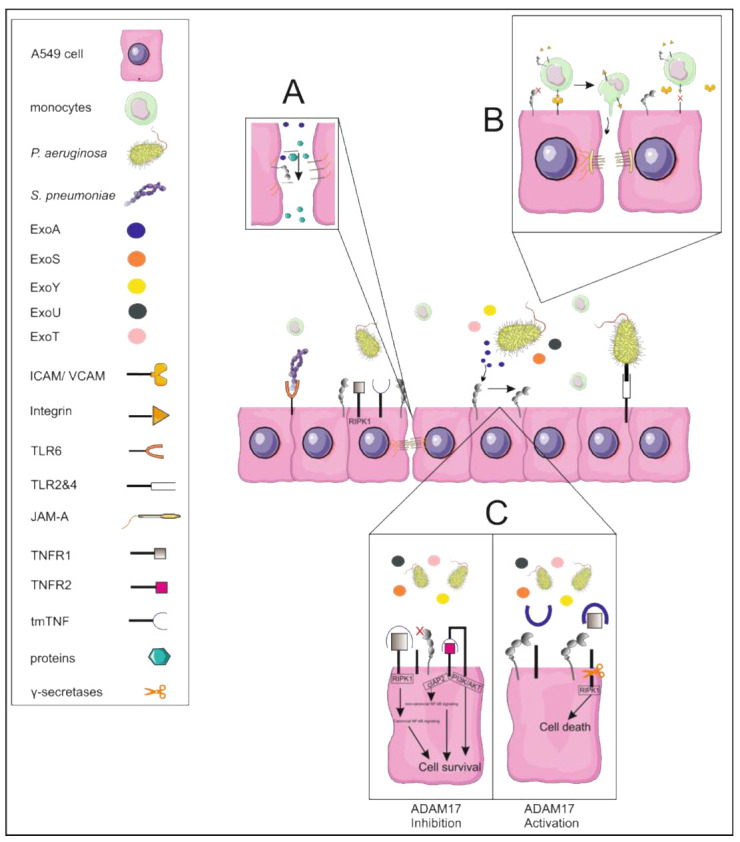
Model of ADAM17 activation during *P. aeruginosa* infection. *S. pneumoniae* and *P. aeruginosa* are recognized through TRL2 and TLR4. ADAM17 activation occurs in a cell-associated manner dependent on the secreted toxin repertoire. Amongst those, ExoA induces the cleavage of junctional adhesion molecules such as JAM-A leading to increased permeability and impairment of regeneration (**A**). Shedding of JAM-A and especially of other adhesion molecules (e.g., VCAM-1, ICAM-1), induced through different exoenzymes (e.g., ExoU), may lead to reduced adhesion of monocytes, limiting the transmigration (**B**). These effects are strongly correlated to reduced lung epithelial cell survival, potentially mediated through TNFR1 shedding (**C**).

*P. aeruginosa* ExoA, as representative of the secreted exoenzymes, is an ADP-ribosyl transferase (ADPRT) that promotes cell death and blocks protein synthesis through inhibition of host elongation factor 2 (EF2) [28]. We observed that inhibition of ADAM17 strongly increased the cell survival of lung epithelial cells during *P. aeruginosa* infection promoting barrier integrity. It was recently shown that ADAM17-mediated shedding of the TNF-receptor 1 (TNFR1) is the initiating event of TNFα-induced necroptosis in endothelial cells promoting tumor cell extravasation and metastasis formation [29]. Similarly, enhanced bacterial phagocytosis by ADAM17-deficient monocytes was partially dependent on cell-autonomous TNFα function [18]. Thus, it is feasible that similar pathways may regulate cell survival in lung epithelial cells (Figure 7A). 

Anti-TNFα therapy has been broadly discussed in the case of Covid-19 therapy (for review see [30]). However, systemic down-regulation may hold a risk for secondary infectious complications. Similarly, long-term inhibition of epidermal growth factor receptor signaling is associated with a higher risk of infections [31] and worsened acute lung injury in murine models [32]. In contrast, inhibition and gene silencing of ADAM17 increased the wound closure upon ExoA treatment and reduced the shedding of JAM-A. The function of JAM-A in tissue cell migration has been controversially discussed. It was shown that JAM-A knockdown in keratinocytes promotes proliferation and wound closure [33]. However, this seems to be limited to single cells in polarized epithelial cells, whereas the presence of JAMA-A is required for collective cell migration [34] (Figure 7B). An increase in full-length JAM-A reflects the decrease of soluble JAM-A, the latter not only limiting tissue cell migration but also transmigration of leukocytes [35]. Indeed, gene silencing and inhibition of ADAM17, respectively, enhanced the transepithelial migration of monocytic cells. Furthermore, we could observe enhanced adhesion of monocytic cells as an initiating event. Besides JAM-A, ICAM-1 and VCAM-1 belong to the substrate spectrum of ADAM17 and have been shown to be expressed and upregulated upon inflammatory stimulation in primary alveolar as well as A459 cells [36,37]. Both adhesion molecules do not only contribute to extravasation, but also to transepithelial migration of monocytes [38]. Thus, through activation of ADAM17 *P. aeruginosa* interferes with the tight regulation of junction and adhesion molecule shedding increasing epithelial dysfunction and limiting monocyte recruitment, which could have a further impact on the clearance capacity (Figure 7C).

ADAM17 may not only function in its cell-associated form but might be also released as soluble ectodomain by the action of ADAM8 resulting in a cell-associated remnant form as was recently shown in breast cancer cells [39]. Furthermore, *P. aeruginosa* triggers the rapid release of ADAM10, the scissoring sister of ADAM17, on exosomes resulting in proteolytic cleavage in trans [12]. Sterile inflammation caused by *E. coli* lipopolysaccharide (LPS) led to the long-term release of ADAM17 on exosomes accompanied by reduced cell-associated expression [40]. In contrast to these observations, we could observe neither degradation/remnant forms of ADAM17 indicating the release of the soluble ectodomain nor a release on exosomes, pointing towards a cell-associated function of epithelial ADAM17 in *P. aeruginosa* infection. Due to the spread of antimicrobial and multi-drug resistance research is more and more focused on the development of novel anti-infectives either interfering with inter-bacterial communication [41] or the released bacterial toxins and their interacting host membrane partners [42]. Indeed, we could show by the use of heat-inactivated bacteria that the observed regulation of ADAM17 is rather dependent on the released toxins than on the particle itself which is in accordance with previous reports on cytokine and cytokine receptor release upon stimulation with *P. aeruginosa* LPS and flagellin infection [43,44]. Nevertheless, we observed a higher activation capacity and stronger functional impacts by living bacteria in comparison to single stimulation with ExoA. Besides ExoA as T2SS-derived exoenzymes, *P. aeruginosa* secreted virulence factors include T3SS-derived toxins (ExoS, ExoT, ExoY, ExoU) and elastase, which are involved in the regulation of lung barrier integrity (for review see [10]). By the use of strains lacking ExoS and ExoT, it was shown that ExoU is the cytotoxin responsible for cell death of lung epithelial cells [45]. Indeed, we observed that ExoA, in contrast to infection with *P. aeruginosa*, had no impact on epithelial cell survival, also explaining the differences in induction of monocyte transmigration. Thus, it is quite feasible that during *P. aeruginosa* infection different toxins contribute to ADAM17 activation and its downstream effects such as induction of protein permeability, regeneration, cell survival, adhesion, and transmigration (Figure 7).

Gram-negative and Gram-positive bacteria are recognized through TLR2 and TLR4 based on their different composition of the cell-wall components [46]. The differential activation of ADAM17 through TLR ligands could be one explanation for the observed differences between *S. pneumoniae* and *P. aeruginosa* but based on our findings the activation of ADAM17 seems to be rather dependent on the toxin repertoire. Pneumolysin, the most prominent secreted virulence factor of *S. pneumoniae*, is a pore-forming toxin, which might activate proteolytic events through an increase in ion conduction [47], for example, for calcium. However, activation of ADAM17 seems to occur in a more kinase-dependent manner, including PKC and ERK/MAPK pathways, with less relevance of calcium influx [48]. ExoS, ExoT, and ExoA display ADP-ribosyltransferase activity, with ExoS and ExoT further containing a RhoGTPase activating domain [49,50]. It has been shown that ADAM17 activation depends on the redox state, for example, changed through the generation of reactive oxygen species involving GTPases [51,52]. Thus, induction of ROS production could be one possible mechanism of ADAM17 activation during *P. aeruginosa* infection. However, the mentioned aspects are speculative so far, stimulating for future studies.

In conclusion, therapeutic targeting of junction and adhesion molecule shedding by ADAM17, limitation of the TNFα-induced cell death or blockage of the activating bacterial toxins may be three novel anti-infective treatment options for *P. aeruginosa*-caused lung infection. We are aware of the fact that this assumption is based on in vitro investigations so far. However, previous in vivo experiments pointed towards a generally pro-inflammatory function of ADAM17 in lung disease. Site-specific inhibition approaches may reduce the induction of vascular leakage [17], increase the phagocytic and clearance capacity of leukocytes [18], prevent the impairment of neutrophil recruitment [53], interrupt the inflammatory relay by smooth muscle cells [54] and reduce epithelial damage. However, further translational studies are required to evaluate the full potential of ADAM17 as an anti-infective therapeutic target in infectious lung disease.

## Figures and Tables

**Table 1:** Concentrations and suppliers of used antibodies and important substances. ADAM, a disintegrin and metalloproteinase; CCL2, chemokine (C-C motif) ligand 2; FITC, fluorescein isothiocyanate; GAPDH, glyceraldehyde-3-phosphate-dehydrogenase; JAM-A, junctional adhesion molecule A; TAPI-1, TNF-alpha protease inhibitor I; TRITC, tetramethylrhodamine; WC, working concentration.

Reagent	Source	WC
rabbit polyclonal anti-human ADAM17 (C-terminus)	Invitrogen (Frankfurt, Germany)	0.1 µg/mL
rabbit polyclonal anti-human GAPDH	Santa Cruz Biotech (Dallas, TX, USA)	0.4 µg/mL
mouse monoclonal anti-human JAM-A (N-terminus)	BD Biosciences (Heidelberg, Germany)	0.25 µg/mL
mouse monoclonal anti-human CD9 (MM2/57)	Invitrogen (Frankfurt, Germany)	1 µg/mL
mouse monoclonal anti-Flotillin-1	BD Biosciences (Heidelberg, Germany)	0.25 µg/mL
Peroxidase-conjugated anti-mouse IgG	GE Healthcare (Chicago, IL, USA)	1:20,000
Peroxidase-conjugated anti-rabbit IgG	GE Healthcare (Chicago, IL, USA)	1:40,000
Human CCL2	Peprotech (Rocky Hill, NJ, USA)	3 nM
TAPI-1	Merck Millipore (Darmstadt, Germany)	10 µM
TRITC-Dextran	Sigma-Aldrich (Taufkirchen, Germany)	1 mg/mL
FITC-Albumin	Sigma-Aldrich (Taufkirchen, Germany)	0.25 mg/mL
Exotoxin A	Sigma-Aldrich (Taufkirchen, Germany)	100 ng/mL

## Data Availability

Not applicable.

## Supplementary Materials

The following supporting information can be downloaded at: https://www.mdpi.com/article/10.3390/cells11152303/s1, Figure S1: Pathogen-specific regulation of ADAM17; Figure S2: Control experiments and JAM-A shedding upon ExoA stimulation.
