# Supplementary material for: Pseudomonas aeruginosa Alters Critical Lung Epithelial Cell Functions through Activation of ADAM17
Source: Cells. 2022 Jul 26;11(15):2303. doi: 10.3390/cells11152303 (PMC9331763; doi:10.3390/cells11152303)
Supplement: Supplementary file 1 [file cells-11-02303-s001.zip › cells-1796010-supplementary.pdf]

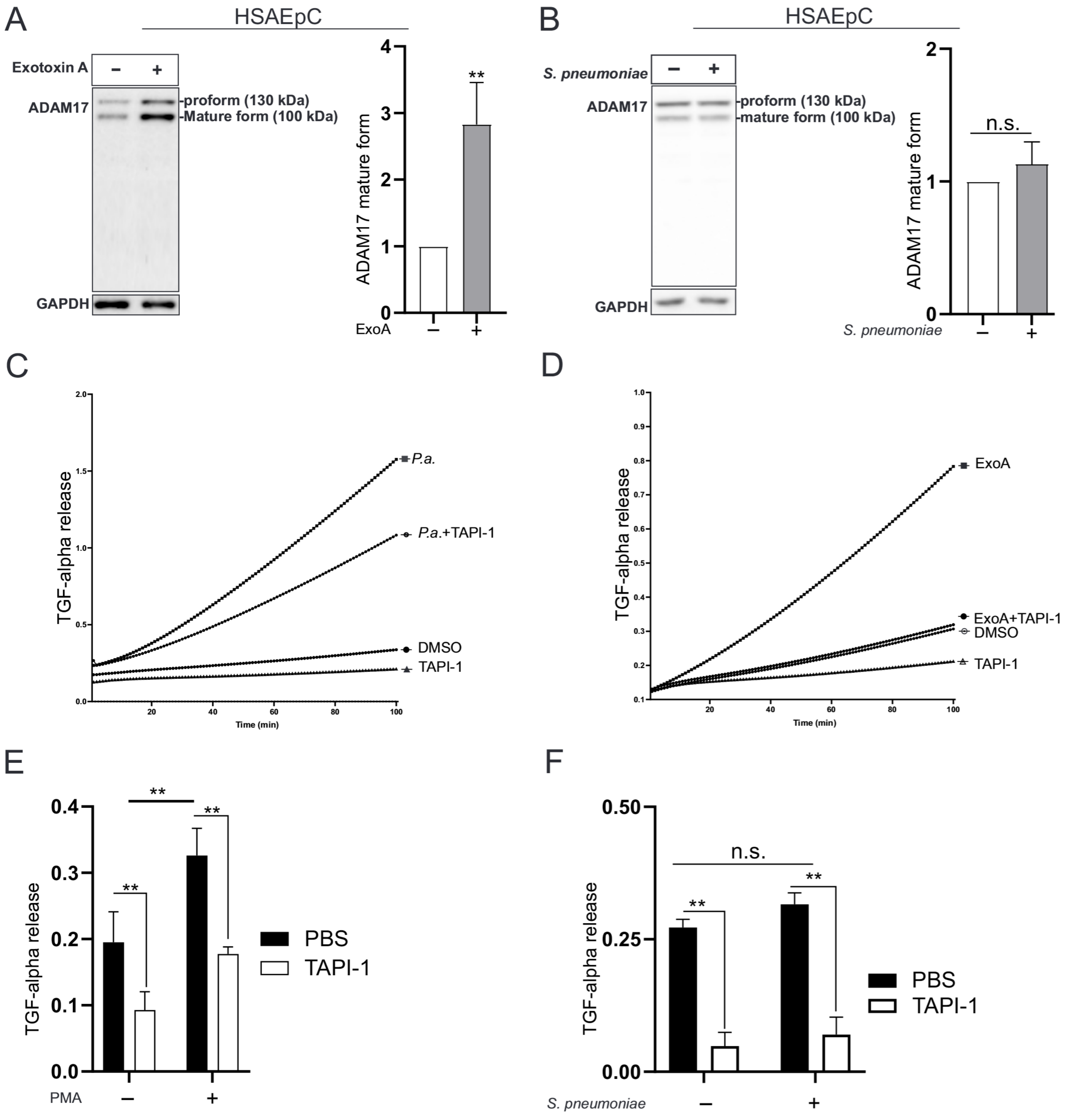

A
HSAEpC
Exotoxin A – +
ADAM17
-proform (130 kDa)
-Mature form (100 kDa)
GAPDH
ADAM17 mature form
**
ExoA – +
B
HSAEpC
S. pneumoniae – +
ADAM17
-proform (130 kDa)
-mature form (100 kDa)
GAPDH
ADAM17 mature form
n.s.
S. pneumoniae – +
C
TGF-alpha release
P.a.
P.a.+TAPI-1
DMSO
TAPI-1
Time (min)
D
TGF-alpha release
ExoA
ExoA+TAPI-1
DMSO
TAPI-1
Time (min)
E
TGF-alpha release
**
PBS
TAPI-1
PMA – +
F
TGF-alpha release
n.s.
**
PBS
TAPI-1
S. pneumoniae – +

Figure S1. **Pathogen specific regulation of ADAM17**. **(A-B)** Human small airway epithelial cells (HSAEpC) were grown until confluence and either left unstimulated, stimulated with ExoA (A, 100 ng/ml) or *S. pneumoniae* (B, MOI 5). Samples were prepared after 4 h of incubation, and the protein expression and maturation of ADAM17 were analyzed by Western blot using an antibody against the C-terminus (intracellular part). The protein expression of GAPDH served as loading control. Band intensities were evaluated by densitometry and normalized to the expression of the unstimulated cells. **(C-F)** A549 cells were transfected with a plasmid encoding for alkaline phosphatase (AP)-coupled TGF-alpha (AP-TGF-α). Cells were pre-incubated for 30 min with TAPI-1 (10 µM) or 0.1% DMSO as a vehicle control and subsequently left unstimulated, infected with *P. aeruginosa* (C, MOI 5 for 4 h) or stimulated with ExoA (D, 100 ng/ml for 4 h), stimulated with PMA (E, 1 µM for 1 h) or infected with *S. pneumoniae* (F, MOI 5 for 4 h). Subsequently, the cells were lysed and the AP activity was analyzed in both the cell lysate and the supernatant as an indicator for TGF-α cleavage and release and calculated as ration of AP-TGF-α activity in the supernatant to the total activity (lysate and supernatant). Representative measurements of AP-TGF-α activity in the supernatant of *P. aeruginosa* infected and ExoA stimulated cell, respectively, are shown in C and D. Quantitative data are shown as means + SD of three independent experiments. Asterisks indicate significance difference to the control calculated using two tailed two samples t-test in A and B and two-way ANOVA and Tukey post-test in E and F (** $p < 0.01$, n.s. not significant).

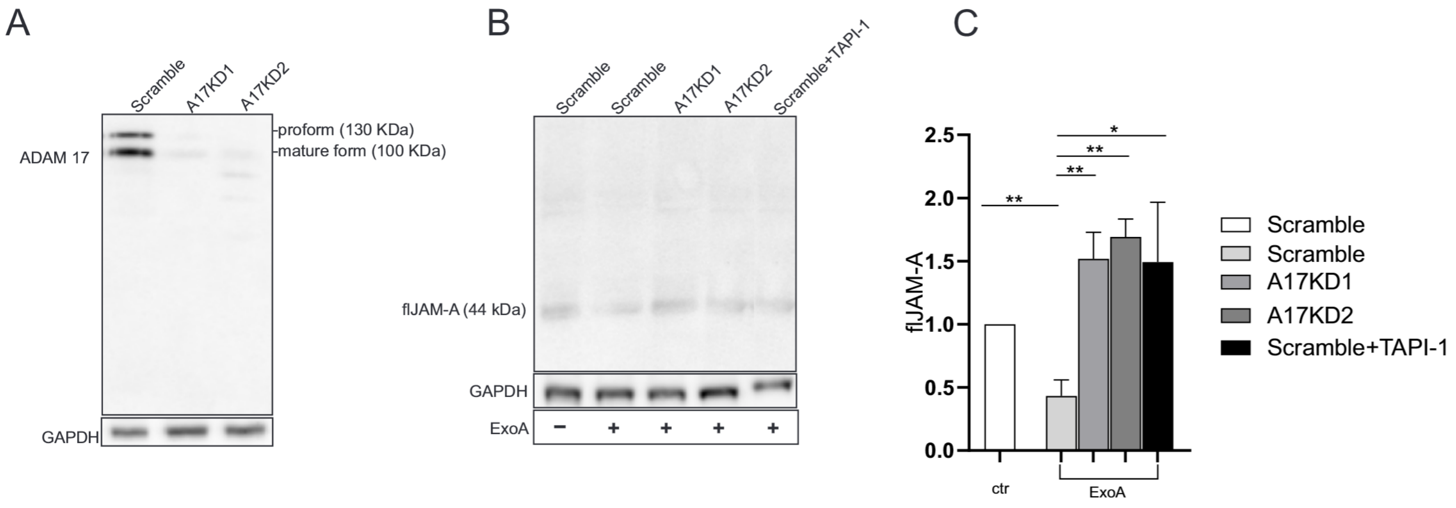
A
Scramble
A17KD1
A17KD2
ADAM 17
proform (130 KDa)
mature form (100 KDa)
GAPDH
B
Scramble
Scramble
A17KD1
A17KD2
Scramble+TAPI-1
flJAM-A (44 kDa)
GAPDH
ExoA
−
+
+
+
+
C
flJAM-A
2.5
2.0
1.5
1.0
0.5
0.0
**
**
**
*
ctr
ExoA
Scramble
Scramble
A17KD1
A17KD2
Scramble+TAPI-1

Figure S2. Control experiments and JAM-A shedding upon ExoA stimulation. A549 cells were transduced with lentivirus encoding shRNA against ADAM17 for knockdown (KD) (A17KD1 or 17KD2) or unspecific shRNA (scramble, scr) as a control. **(A)** One week after transduction, the protein expression of ADAM17 was analyzed by Western blot using an antibody against the C-terminus (intracellular part). The protein expression of glyceraldehyde-3-phosphat dehydrogenase (GAPDH) served as loading control. **(B/C)** Cells were either left unstimulated or stimulated with Exo A (4 h, 100 ng/ml). Cells were lysed and the expression of JAM-A was analyzed by western blot using an antibody against the N-terminus (extracellular part). GAP DH served as a loading control. Band intensities were evaluated by densitometry and normalized to the expression of the unstimulated cells. A representative blot is shown in B. Quantitative data are shown as means + SD of three independent experiments. Asterisks indicate significance difference to the control calculated using two tailed two samples t-test (* $p < 0.05$, ** $p < 0.01$).
